# Differential efficacy of first licensed western vaccines protecting without immunopathogenesis Wuhan-1-challenged hamsters from severe COVID-19

**DOI:** 10.1038/s41541-025-01100-5

**Published:** 2025-03-17

**Authors:** Aileen Ebenig, Mona V. Lange, Michelle Gellhorn Serra, Alexandra Kupke, Roland Plesker, Bingqian Qu, Richard J. P. Brown, Thorsten J. Maier, Michael D. Mühlebach

**Affiliations:** 1 Division Veterinary Medicine, Paul-Ehrlich-Institut, 63225 Langen, Germany; 2 Institute for Virology, Philipps University, 35043 Marburg, Germany; 3 German Center for Infection Research, Gießen-Marburg-Langen, Germany; 4 Division Safety of Biomedicines and Diagnostics, Paul-Ehrlich-Institut, 63225 Langen, Germany; 5 Present Address: Department of Translational and Computational Infection Research, Ruhr University Bochum, 44801 Bochum, Germany

**Keywords:** Viral infection, Experimental models of disease, Vaccines, Vaccines, Adaptive immunity

## Abstract

Four COVID-19 vaccines were developed, tested, and authorized early in Europe and the US. Comirnaty and Spikevax are mRNA-based, whereas Jcovden and Vaxzevria utilize adenoviral vectors (AdV). We described a hamster model of COVID-19 utilizing Wuhan-1 strain SARS-CoV-2, in which vaccine-associated immunopathogenesis can be induced by Alum-adjuvanted Spike protein (Alum+S). Such animals were vaccinated with the authorized vaccines or Alum+S, challenged, and examined. All vaccinated hamsters produced antibodies targeting S. Neutralizing antibodies (nAb) were induced only by authorized vaccines. While nAbs were present after one vaccination with AdV-vaccines, mRNA vaccines needed a boost immunization. Upon challenge, all authorized vaccines protected from severe disease. Less tissue damage and no live virus (one exception) were detectable in the lungs. In contrast, Alum+S immunized hamsters developed VAERD. Our data reveal the absence of induction of VAERD by early commercial vaccines in hamsters, while animals´ immune responses and protection seem to match the clinical vaccine efficacy.

## Introduction

To combat the COVID-19 pandemic caused by the severe acute respiratory syndrome coronavirus 2 (SARS-CoV-2), a number of different vaccines have been developed, tested, and licensed in a remarkably short time. To date, more than 13 billion doses (March 12, 2024^1^) of those authorized vaccines have been administered. These vaccines were developed on the basis of traditional approaches such as inactivated virus or adjuvanted, recombinant proteins, but especially in the Western World by novel technologies, i.e., mRNA vaccines or adenoviral vectors. Remarkably, all of the latter target the Spike protein S of SARS-CoV-2, which is the target of neutralizing antibody responses due to its role in receptor binding and cell entry during the pathogen life cycle.

One potential safety risk in vaccine development is the enhancement of the disease in vaccinated individuals later infected with the respective pathogen due to the induction of immune responses that facilitate the development of immunopathologies. Enhancement of disease by vaccine-induced syndromes such as vaccine-associated enhanced respiratory disease (VAERD) or antibody-dependent enhancement (ADE) has been described for a number of different viral diseases such as infections with dengue virus^2^, respiratory syncytial virus^3–6^, or feline infectious peritonitis virus^7–9^. Accordingly, animal models revealed the potential risk of VAERD for infections with the first two highly pathogenic human beta-CoVs, SARS-CoV, or Middle East respiratory syndrome (MERS)-CoV. After superinfection of receptor-transgenic mice previously immunized with whole-inactivated virus, the animals developed severe lung pathology with massive infiltration of eosinophils^10–12^. Such enhanced immunopathology has been linked to the induction of low-affinity and non-neutralizing antibody responses^6,7^ or vaccine-induced T_H_2-biased T cell responses^5^.

Although such enhancement processes have not been described for SARS-CoV-2 in human vaccinees, typical features of VAERD have been observed in T_H_2-biased mice vaccinated with alum-adjuvanted whole-inactivated virus or S protein upon subsequent infection with mouse-adapted SARS-CoV-2^13,14^. Similarly, we detected and proposed a detailed mechanism for the induction of VAERD in outbred hamsters vaccinated with Alum-adjuvated S protein upon subsequent infection with a low-passage human patient isolate of SARS-CoV-2. Our model suggests that the induction of a T_H_2-specific S-specific T cell response and the induction of low levels of non-neutralizing antibodies leading to Fc-receptor-mediated uptake of the virus into lung macrophages are the main causes of the development of VAERD^15^. Absence of such reactions may be attributed at least in part by the design of most of the authorized vaccines the construction of which considered this potential risk by avoiding induction of T_H_2-biased immunity^16–22^.

Here, we have utilized our hamster model of severe COVID-19^23^ to analyze side-by-side the protective effects of the four vaccines, which received early approval in the European Union and the United States: Comirnaty (BioNTech)^19^ and Spikevax (Moderna)^16,17^ are based on mRNA vaccine technology while Jcovden (Johnson & Johnson)^20–22^ and Vaxzevria (AstraZeneca)^18^ utilize the adenovirus vector (AdV) platform technology. To our knowledge, our study presented here is the first comparing all four commercial vaccines derived from two very different vaccine platform technologies in a relevant animal model for COVID-19, simultaneously, allowing assessment of vaccine efficacy and potential adverse events side-by-side.

All four vaccines protected the hamsters after twofold vaccination from severe disease induced in the animals by an early SARS-CoV-2 isolate of the Wuhan-1 lineage without showing evidence of eosinophil infiltration as a hallmark of immunopathogenesis. Only one out of 16 hamsters had low titer infectious virus in the lungs, while viral RNA copy numbers were around the limit of detection in lungs and BAL fluid. This protection correlated with the induction of considerable nAb responses by all tested authorized vaccines. As a remarkable difference between vaccine concepts, both AdV-derived vaccines induced nAb titers already after one vaccination, while the second vaccination with the mRNA vaccines fully compensated for this initial difference.

## Results

### Protection by authorized COVID-19 vaccines in the immunopathogenesis-prone hamster infection model

**Fig. 1:**
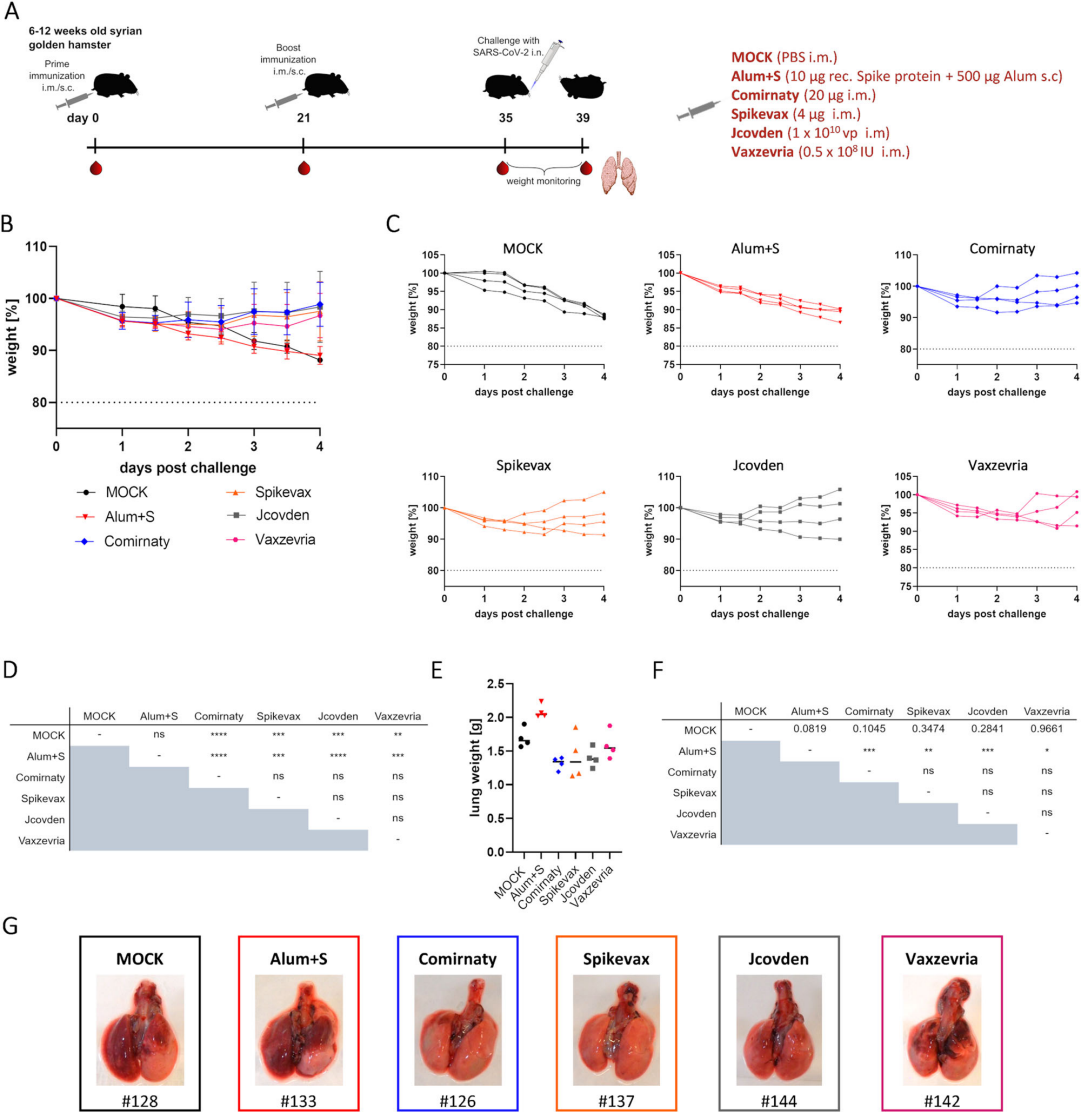
Protection of Syrian Hamsters by different vaccines. **A** Schematic depiction of a challenge experiment in vaccinated Syrian golden hamsters. Six to 12-week-old Syrian hamsters were immunized with indicated vaccines on days 0 and 21. On day 35, immunized hamsters were intranasally challenged with low-passage SARS-CoV-2 and observed for 4 days before sacrifice and organ preparation. Blood was drawn as indicated on days 0, 21, and 35 for analysis of humoral immunity. **B**, **C** Evidence of protection by reduced weight loss. Depicted are weight curves of **B** means of hamster cohorts (*n* = 4) with indicated immunizations (error bars indicate SD) or **C** individual animals in these groups. **D** Statistic analysis of groups depicted in (**B**). For statistical analysis, two-way ANOVA was applied with Tukey’s multiple comparisons test. ns not significant, **p < 0.01; ***p < 0.001; ****p < 0.0001. **E** Weight of hamster lungs on day 4 after SARS-CoV-2 challenge exemplarily depicted by lungs of indicated hamsters in (**G**) revealing macroscopic lung pathology. **F** Statistical analysis of groups depicted in (**E**). For statistical analysis, ordinary one-way ANOVA was applied with Tukey’s multiple comparisons test. *p < 0.05; **p < 0.01; ***p < 0.001.

Groups of six to 12-week-old Syrian golden hamsters (*n* = 4) were immunized side-by-side with adapted doses of authorized COVID-19 vaccines Comirnaty, Jcovden, Spikevax, or Vaxzevria on days 0 and 21 via the intramuscular (i.m.) route (Fig. 1A). To control for inducibility of immunopathogenesis, recombinant unmodified, not stabilized SARS-CoV-2 Spike protein adjuvanted with aluminum hydroxide gel (Alum+S) was injected subcutaneously in a separate cohort by the same schedule, as described before^15^. Applied doses were chosen in accordance with previously published literature for mice and hamsters presumably preparing clinical trials to ensure proper immune reactions against all vaccines in this animal model. Medium-inoculated hamsters served as naïve controls (MOCK). Two weeks after the second vaccination, all hamsters were challenged intranasally (i.n.) with a low-passage SARS-CoV-2 patient isolate. Animals were monitored for 4 days, sacrificed, and lungs were prepared for histopathology, analysis of viral RNA copy numbers, and titration of live virus.

As previously described, hamsters immunized with Alum+S were not protected and lost weight upon infection with the same kinetics as naïve control animals (Fig. 1B, C). Moreover, the mean weight of the lungs (2.088 g) was increased as compared to naïve animals (1.694 g), indicating immune cell infiltration and exaggerated inflammatory processes, as also suggested by gross macroscopic analysis of the lungs (Fig. 1E, G). In contrast, most animals in all vaccine cohorts recovered after initial weight loss after day 2 post infection. Lung weights were significantly reduced compared to the Alum+S cohort (Fig. 1E, F) and nearly reached significance for the Comirnaty group also in comparison to naïve infected animals. Comirnaty, Jcovden, or Spikevax-immunized animals had comparable mean lung weights between 1.319 g to 1.417 g, while the Vaxzevria cohort revealed a slightly higher mean lung weight of 1.589 g, although this difference did not reach statistical significance. These observations came along with reduced pathological alterations of lungs on the macroscopic (Fig. 1G and Suppl. Fig. S1) as well as microscopic levels (Suppl. Fig. 3A).

**Fig. 2:**
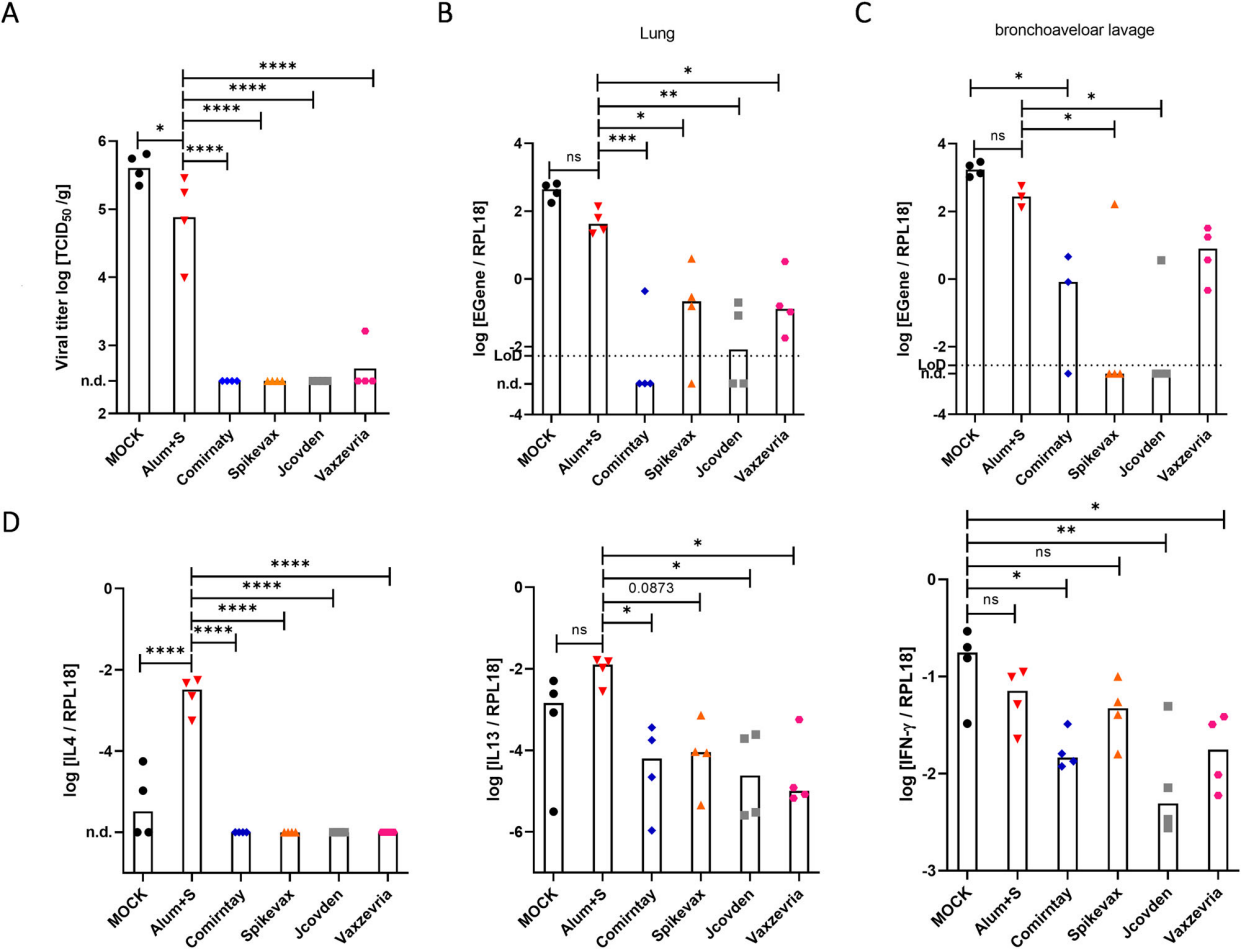
Protection by reduction of organ virus load. Virus load in infected hamsters determined (**A**) by titration of live virus titers in lung tissue or **B**, **C** quantification of viral RNA by qRT-PCR in **B** lung tissue or **C** bronchoaveloar lavage (BAL) 4 days post infection. **D** Quantification of IL-4 (left), IL-13 (middle), and IFN-γ (right) expression relative to RPL18 housekeeping gene by qRT-PCR of mRNAs to determine the bias of reactive T cells in vaccinated animals after SARS-CoV-2 infection. Single data points indicate individual animals. The horizontal bar denotes the median of each group. For statistical analysis, ordinary one-way ANOVA was applied with Tukey’s multiple comparisons test. ns not significant (*p* > 0.05), *p < 0.05; **p < 0.01; ***p < 0.001; ****p < 0.0001.

In these animals, nearly no live virus was detected in lung tissues, except for one animal from the Vaxzevria group with a low titer of 1.6 × 10^3^ TCID_50_/g (Fig. 2A). However, also hamsters immunized with Alum+S revealed lower virus loads in the lung compared to naïve, infected mock animals (Fig. 2A), again indicating the immunopathological mechanism of disease in these animals. Analysis of relative abundance of viral RNA in lung tissue (Fig. 2B) and bronchoalveolar lavages (Fig. 2C) were in accordance with live titers: In the cohorts, which had been vaccinated with Comirnaty or Jcovden, at least half of the analyzed samples were below the limit of detection. Three out of four animals immunized with Spikevax had low, but detectable viral RNA load (median 0.227 E/RPL18 gene copies). Only in the Vaxzevria cohort, all samples revealed a low, but detectable RNA load about 500-fold lower than naïve control animals (median 0.134 vs. 458.17 E/RPL18 gene copies). In contrast, Alum+S immunized animals also had a lower RNA load (median 45.61 E/RPL18 gene copies) detectable in all animals, but the reduction was just about tenfold.

To also characterize the bias of antigen-specific T cell responses induced by vaccination, we analyzed mRNA expression profiles of IL-4, IL-13, or IFN-γ in the lungs of SARS-CoV-2 infected animals. Absolute mRNA copy numbers were determined and compared to the RPL18 housekeeping gene. IL-4 mRNA was found in lung samples from hamsters vaccinated with Alum+S as well as in two vaccination-naive infected animals, albeit to a lesser extent than in the Alum+S group. No IL-4 expression was evident in samples from hamsters immunized with any of the authorized vaccines. The highest abundance of IL-13 mRNA was also observed in the Alum+S immunized cohort, followed by naive infected animals. Significantly lower expression of IL-13 was found in the majority of hamsters immunized with all authorized vaccines. In contrast to those cytokines indicating a T_H_2-biased immune response as evident in the Alum+S vaccinated animals, IFN-γ expression was comparable in all animals, but highest in the vaccination-naïve cohort, and roughly correlated with remaining virus loads (Fig. 1D).

### In situ hybridization (ISH) indicates active SARS-CoV-2 infection in tissues with pathological alterations

**Fig. 3:**
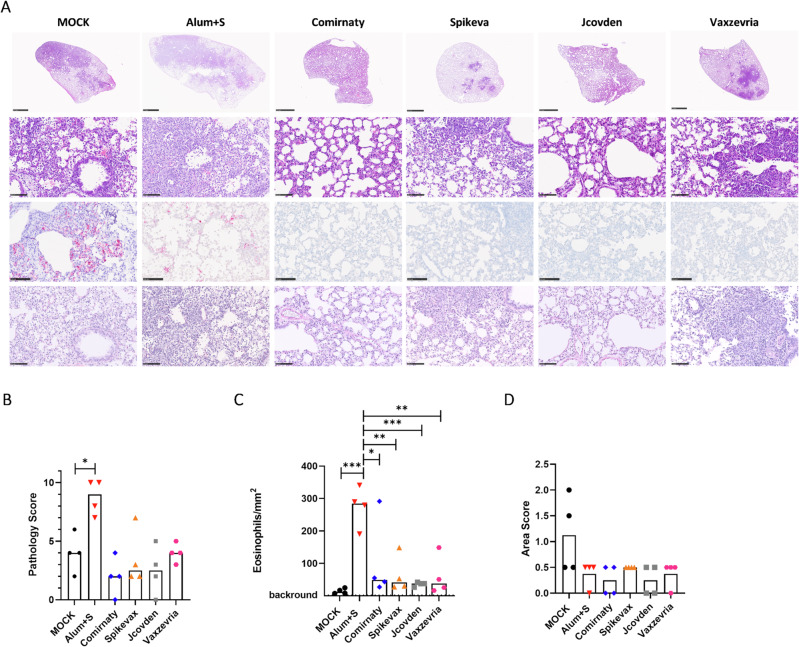
Absence of immunopathogenesis and modulated pathology in vaccinated hamsters. Depicted hamster lungs were formalin-fixed, embedded in paraffin, and tissue slices were stained with hematoxylin-eosin. The protective efficacy of vaccination was analyzed by **A** histopathology of lung sections prepared 4 dpi (scale bars: top row, 2.5 mm; all other rows, 100 µm). **B** HE stained slices were assessed blinded by a veterinary pathologist and scored for dense tissue areas with immune infiltration, inflammation of vessels, and abundance of eosinophils as a marker of VAERD. The resulting scores are indicated. Single data points indicate individual animals´ samples. The bar denotes the median of each group. For statistical analysis, ordinary one-way ANOVA was applied with the Mann–Whitney test. ns, not significant; *p < 0.05. **C** Eosinophil quantification for all animals is depicted. Eosinophil numbers in ten randomly selected fields of view at 200x magnification were counted, and means were correlated against the area of view. Each data point reveals the mean number of eosinophils per mm^2^ in individual animals. For statistical analysis, ordinary one-way ANOVA was applied with Tukey’s multiple comparisons test. *p < 0.05; **p < 0.01; ***p < 0.001. **D** Infected areas positive by ISH against SARS-CoV-2 viral RNA were quantified by determining the relative area of tissue staining positive for viral RNA by automated image evaluation of the whole slice.

As already indicated by the detection of viral RNA by qRT-PCR, active SARS-CoV-2 infection in the lungs of the infected animals was demonstrated by ISH of SARS-CoV-2 RNA genomes in lung tissue (Fig. 3A, third row and 3D). As previously reported, Alum+S vaccinated, infected animals showed reduced staining for SARS-CoV-2 RNA genomes (mean area score 0.375) as compared to infected mock-immunized hamsters (mean area score 1.125). All four vaccine cohorts showed reduced staining for SARS-CoV-2 RNA genomes compared to naïve, infected animals to a different extent (mean area scores: Comirnaty 0.25; Jcovden 0.25; Spikevax 0.5; Vaxzevria 0.375). Interestingly, only in the Spikevax group, all animals had detectable area scores for SARS-CoV-2 RNA genomes.

### Absence of immunopathogenesis upon immunization with authorized vaccines

In accordance with our previous report^15^, histological analysis of hematoxylin and eosin (H&E)-stained lung tissue slices revealed the replication of the expected pathology for the control animals. In naïve, infected hamsters, moderate inflammation of the epithelia and endothelia in bronchi and vasculature was observed (SI-Table S1). Moderate infiltration of macrophages and lymphocytes resulted in a fraction of 1% to 50% of dense areas (SI-Table S1). Among the immune cell infiltrates, no or few eosinophils or granulocytes were observed, with the exception of a single animal showing significant infiltration of granulocytes. Individual foci of moderate karyorrhexis became also evident in these animals. In contrast, Alum+S-vaccinated, infected hamsters revealed an intensified pathology (Fig. 3A, B). The areas of dense infiltrates varied between 20 and 50% of the tissue slices (Suppl. Fig. S2 and SI-Table S1). Large areas of inflammation of bronchi and vasculature included massive infiltration of eosinophils (Fig. 3C).

On the other hand, lung tissue derived from hamsters vaccinated with mRNA-based vaccines Comirnaty or Spikevax revealed reduced inflammation, with only 0% to 10% of the tissue becoming dense in most animals (SI-Table S1). Only a single hamster, each, immunized either with Comirnaty or Spikevax, exhibited 25% and 30% dense areas, respectively (SI-Table S1). Most animals revealed low to no eosinophilic infiltrates in the lungs (Fig. 3C). Thus, the pathological score was considerably reduced for both groups, with one animal in the Comirnaty group scoring as completely “healthy” (Fig. 3B). Hamsters vaccinated with AdV-based vector vaccines Jcovden or Vaxzevria revealed 1 to 40% dense areas. Interestingly, three out of four animals in the Jcovden group had between 0 and 5% dense areas, again one animal scoring “healthy” (Fig. 3B). However, another Jcovden hamster had up to 40% dense areas. On the other hand, three out of four animals receiving Vaxzevria had between 20% and 40% dense areas. Here, a single animal in this group had only about 1% compacted tissue. Again, no to low numbers of eosinophils were observed in the lung tissue (Fig. 3A).

Sirius Red staining for eosinophils confirmed significant infiltration of eosinophils in infected hamsters inoculated with Alum+S, as already observed in H&E-stained lung sections. In these animals, between 190 and 340 eosinophils/mm^2^ (mean 275.1 eosinophils/mm^2^) were found. In contrast, the mean number of eosinophils was reduced 2.6- to 7.6-fold (mean: 35.9–104.3 eosinophils/mm^2^) in hamsters vaccinated with the authorized COVID-19 vaccines. Only one animal from each, the Comirnaty (291.6 eosinophils/mm^2^), Spikevax, or Vaxzevria group (both 148.8 eosinophils/mm^2^) had a higher eosinophil density (Fig. 3C).

Thus, these data confirm the protection of immunized animals in the absence of any signs of VAERD in hamsters vaccinated with the authorized COVID-19 vaccines, in a severe COVID-19 hamster infection model capable of revealing pathological alterations indicative of vaccine-induced immunopathogenesis.

### Protection correlates with induction of nAbs, immunopathogenesis with lower titers of bAbs in the absence of nAbs

**Fig. 4:**
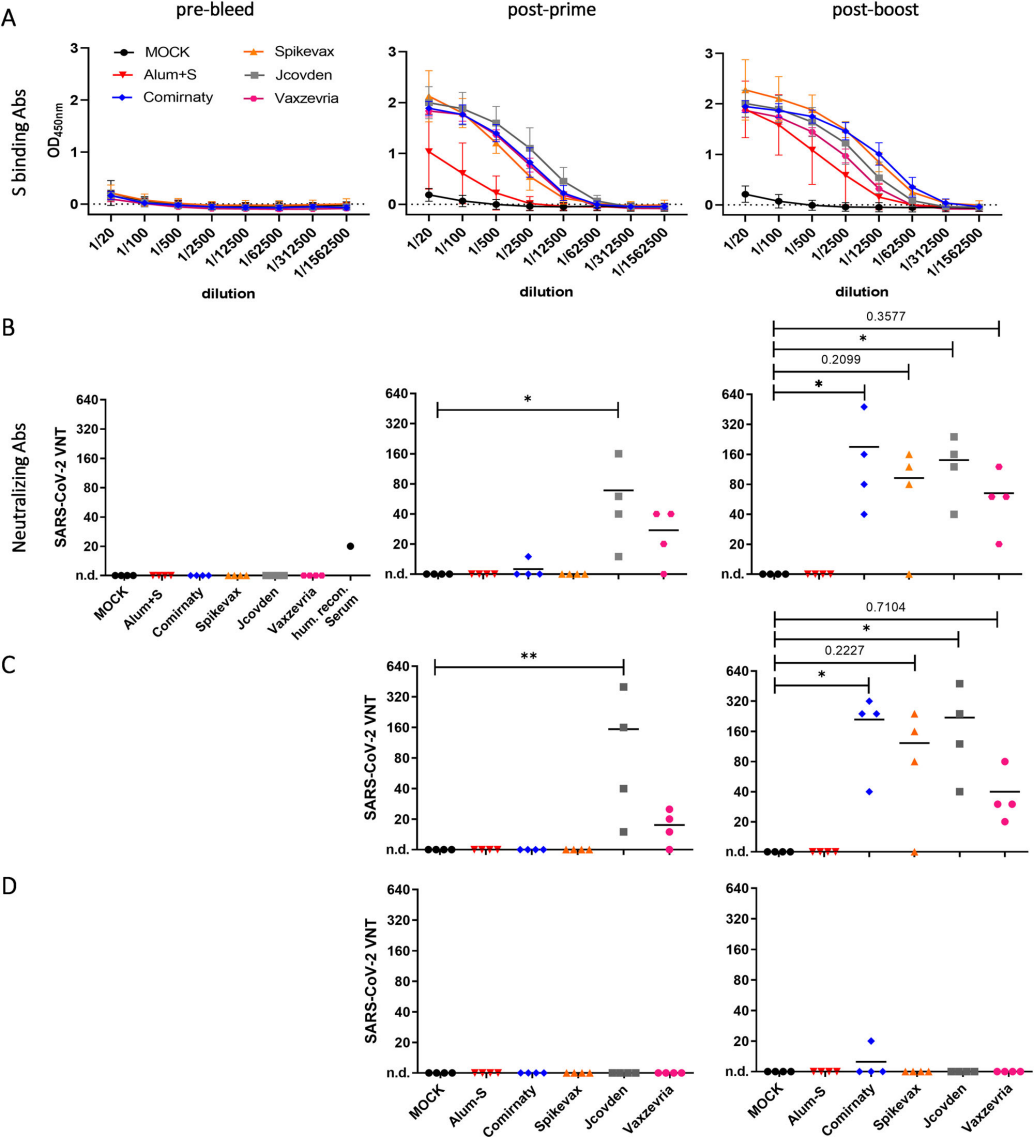
Induction of SARS-CoV-2 Spike-specific antibodies in vaccinated hamsters. Sera of immunized hamsters sampled on days 0 (pre-bleed), 21 (post-prime), and 35 (post-boost) were used for determination of binding and neutralizing antibodies by **A** ELISA or **B** titration of neutralizing Abs by microneutralization test. Depicted are **A** means and the respective standard deviations of groups (*n* = 4) or **B**–**D** titers of individual animals (horizontal bars indicate mean) against **B** SARS-CoV-2 Wuhan virus (Muc-IMB1), **C** Delta VOC (B1.617.2), or **D** Omicron VOC (B.1.1.529). Virus-neutralizing titers (VNT) are reciprocals of the highest dilution, abolishing infectivity. For statistical analysis, the Kruskal–Wallis test was performed in combination with Dunn’s multiple comparisons test, *p < 0.05.

Fourteen days after the second immunization, sera were analyzed for binding antibodies (bAbs) targeting S by ELISA in comparison to pre-vaccination (“pre-bleed”) and post-prime sera. Binding antibodies were induced after the primary vaccination for all vaccine groups and boosted by the second immunization for the mRNA vaccines but only moderately for the AdV-based vaccines. In contrast, no SARS-CoV-2 Spike bAbs were detected in pre-bleed sera or sera derived from MOCK-immunized animals. All authorized vaccines induce comparable amounts of SARS-CoV-2 Spike bAbs. In contrast, the experimental Alum+S vaccine induced lower amounts of bAbs in comparison to the authorized vaccines. (Fig. 4A).

We also determined neutralizing antibody responses to SARS-CoV-2 Muc-IMB1, by virus neutralization test (VNT) (Fig. 4B). Neutralizing antibodies (nAb) were detected in sera of all hamsters that were immunized with authorized vaccines after boost immunization, whereas hamsters that received Alum+S showed only S-binding antibodies without neutralizing activity, as previously reported^15,23^. Uniform induction of nAbs by mRNA vaccines was observed after the second dose; only serum from a single animal in the Spikevax group lacked detectable nAb titers after the boost immunization. On the other hand, a single hamster in the Comirnaty group showed low nAb titers already after a single dose of the vaccine. Vaccination with the AdV-based vaccines induced nAb in most animals (4/4 for Jcovden and 3/4 for Vaxzevria) already after prime immunization, but titers increased only marginally after the second shot. Nevertheless, also the fourth Vaxzevria animal became positive for nAbs after the boost immunization. Overall, nAb titers appeared to be slightly lower in animals vaccinated with Vaxzevria compared to those immunized with the other authorized vaccines (Fig. 4B). Similar results were obtained when determining nAb titers against SARS-CoV-2 Delta variant (B1.617.2). While mRNA-based vaccines uniformly required a second booster dose to develop nAbs, AdV-based vaccines had measurable nAb titers already after prime, and also the patterns of individual animals´ reactivity remained (Fig. 4C). In contrast, no nAb titers against the Omicron variant B.1.1.529 were observed in the majority of all vaccinated animals. Only one hamster immunized with Comirnaty revealed low nAb titers after the boost immunization (Fig. 4D).

Taken together, our data show that VAERD does not occur in SARS-CoV-2 infected hamsters immunized with all licensed vaccines tested, due to the absence of eosinophils in tissue sections in the context of lack of T_H_2-bias of S-specific T cell immunity, a marker for VEARD. All vaccines tested significantly reduced viral load in the lung, and no significant differences were observed between the different vaccine cohorts. However, animals vaccinated with Vaxzevria appeared to have a slightly higher lung weight and viral load compared to the other three vaccine cohorts, but still reduced when compared to the naïve, infected hamsters.

## Discussion

During the COVID-19 pandemic, billions of vaccine doses have been administered, and to date, VAERD has not been described in vaccinees. However, the potential risk of the induction of VAERD for SARS-CoV-2 and related Corona viruses has been described in different animal models, and induction of VAERD has been linked to a T_H_2-biased immune response^10–15,24^. Although the transferability of these findings to human beings remains unclear, all vaccines authorized in the European Union and the United States were constructed to elicit a predominantly T_H_1-biased immune response to exclude the risk of potential induction of VAERD^16–21^. These vaccines have previously shown effectiveness in mice^16,25–27^, Non-human primates^17,21,27–30^, some in Syrian hamsters^21,31–34^ and in clinical trials^18–20,22,35–37^. Syrian hamsters infected with SARS-CoV-2 consistently reveal the characteristics of severe COVID-19 and are considered as gold standard for reflecting pathogenesis^38^. Furthermore, Syrian hamsters show, under specific conditions, VAERD upon vaccination with T_H_2-biased vaccines^15^ in the absence of genetic alterations required for either mice^39^ or virus^40^ as necessary in the mouse model, but confirming pathologies observed in those models.

Hence, we tested all four early approved western vaccines side-by-side in this animal model and confirmed the absence of VAERD in SARS-CoV-2 infected hamsters vaccinated with these vaccines, while respective control hamsters (Alum + S) developed VAERD. Consistent with our previously published analysis, T cell cytokine expression patterns revealed induction of T_H_2-biased responses by Alum+S^15^, but the absence thereof in the animals vaccinated by the authorized vaccines. Therefore, our data indicate that the absence of reports of VAERD in previously vaccinated western patients after COVID-19 superinfection are indeed due to the appropriate design of these vaccines and not due to the close similarity of VAERD pathologies and symptoms during severe courses of COVID-19^41^. However, our study is limited to mRNA and AdV-derived vaccines available at the time of initiation of our study, which have been used in billions of immunizations. Unlike the licensed mRNA vaccines, which utilize the S protein stabilized by few mutations in the pre-fusion conformation, the unmodified S protein was used in the Alum+S vaccine in this study, which may be one factor to explain the lower induction of especially nAbs. The performance of other vaccine concepts, e.g., Alum-adjuvanted, inactivated COVID-19 vaccines approved for emergency use by the WHO^42^, needs to be analyzed in future additional studies.

Besides demonstrating the safety of all tested vaccine concepts as the primary study end-point, our data allow comparison of the efficacy of the different vaccines in the same setting. Although all commercially available vaccines tested in our study were protective, as they had demonstrated before in individual pre-clinical and clinical studies, minor differences between the vaccine cohorts were observed during our experiment, although not statistically powered for those analyses at the start. Hamsters which received the AdV-based vaccines showed only a low increase in binding antibodies by the second immunization while neutralizing antibody titers that were induced already after one shot were boosted. This effect has been previously observed for Jcovden^21^, also in the hamster model. In contrast, a two-dose regime of Jcovden boosted binding and neutralizing antibody titers in non-human primates^30^. Those differences were attributed to species-specific effects of the responses against the AdV vaccine backbone after the first immunization affecting the immunogenicity of the boost^21^. In contrast, all hamsters vaccinated with an mRNA vaccine showed strong boosting of binding and neutralizing antibody titers by the second vaccine dose in our study, which confirms previous data^19^. Boosting of binding antibody titers by repeated vaccination with mRNA vaccines has already been described in hamsters vaccinated with 5 μg Spikevax twice^34^. Interestingly, only the serum of a single animal immunized with Comirnaty showed a low neutralizing capacity against SARS-CoV-2 after one shot, in our study, while 7/8 animals among the mRNA vaccine groups developed neutralizing antibodies after twofold immunization. This is somewhat consistent with previous data providing evidence that not all (3/15) hamsters immunized with Spikevax developed neutralizing antibodies after prime immunization^34^. In the end, final nAb titers were comparable to those in animals immunized with AdV-based vaccines, showing no clear advantage for any vaccine technology in a prime-boost regimen concerning humoral responses, while the AdV technology seems advantageous for a one-shot regimen. When analyzing the cross-neutralization of evolved variants of concern by those hamster sera, we noticed that the Delta virus was still neutralized with the same pattern as the original homologous isolate, while the Omicron virus was only weakly neutralized by one single serum, as expected from clinical data testing this immune evasion lineage^43,44^.

Anyway, all authorized vaccines were protecting against the Wuhan lineage challenge virus in our hamster model without evidence for immunopathologies, consistent with clinical findings and protection of human vaccinees. The live virus was recovered from the lungs of only a single Vaxzevria- vaccinated animal close to the limit of detection, but not in any other AdV- or mRNA-vaccinated hamster. The absence of live, replicating virus in the vast majority of animals at this time-point corroborates our chosen schedule for analyses. VAERD induction occurring at later time-points appears thus highly unlikely in the absence of virus- and antigen-production in situ. Previously, the live virus was detected in the lungs of 3 out of 12 Jcovden-vaccinated animals 4 days after infection with 10^2^ TCID_50_ SARS-CoV-2 G614^21^. While our smaller cohort size may explain this difference in the detection of underrepresented animals with lower protection, we detected viral RNA in the lung tissue of two out of four animals vaccinated with Jcovden twice, providing some evidence for initial infection. In any case, vRNA copies in these hamsters’ lungs were significantly lowered, consistent with protection. Also for Spikevax-immunized hamsters, no live virus was found in the lungs after infection, before^34^. Again, viral RNA in lung tissue of mRNA-immunized hamsters was detectable in the Comirnaty- (1/4) or Spikevax-cohorts (3/4). The absence of live virus also in the individual Spikevax hamster without detectable nAb titers suggests that SARS-CoV-2 specific T cell responses induced by the vaccines play a role in protection against infection in our hamster model. Accordingly, mRNA-vaccines protected B cell-depleted vaccinees. Despite the lack of SARS-CoV-2-specific Ab titers, the induced T cell responses reduced the risk to develop severe COVID-19^45,46^. Moreover, distinct subpopulations of CD8^+^ T cells were identified 28 days after immunization with Comirnaty. Frequency and differentiation of the same T cells correlated with preferable clinical outcomes in COVID-19 patients, thus hinting at their importance for protection^47^. Our data provide also evidence for induction of S-specific T cells in our hamster model, as well, since the expression of distinct T cell cytokines after infection is modulated according to the vaccination status.

While our experiments confirmed the VAERD animal model and protection by the authorized vaccines, they also allow direct comparison of the immunogenicity of all four early approved COVID-19 vaccines. Most interesting, the presence of nAb responses after a single immunization as observed by the AdV vectored vaccines are well reflected by the initial authorization of Jcovden as single-shot vaccine^22,48^. Consistent with our hamster data, the comparison of humoral immune responses in human vaccinees who received Spikevax, Comirnaty, or Jcovden nevertheless revealed evidence that titers of SARS-CoV-2 binding and neutralizing antibodies induced by a single immunization with Jcovden was significantly lower than the titers observed for the two mRNA vaccines applied in a prime-boost schedule^49^. However, also homologous boosting increases antibody titers in humans immunized with Jcovden^50,51^, reaching just slightly lower titers than individuals who had received two doses of Comirnaty^50^, as also found in our experiments, here.

In summary, our hamster study confirms the efficacy of all tested vaccines in the absence of VAERD. Thereby, the rather small differences observed in immunogenicity between the early authorized vaccines recapitulate observations in vaccinated human populations and further confirm the validity of the Syrian golden hamster model for COVID-19 vaccine research and analysis, but direct transfer between animal model and human patients needs careful analysis, also in the future. Nevertheless, such animal models, which effectively recapitulate human disease and immunopathology phenotypes, are still needed due to the constant emergence of new dominant SARS-CoV-2 variants such as the Omicron sublineage. These established models can be rapidly repurposed to validate the adaption of existing vaccine platforms or for the development of novel vaccine concepts.

## Methods

### Cells

Vero clone E6 was purchased from ATCC (ATCC# CRL-1586, Manassas, VA, USA) and cultured in Dulbecco’s modified Eagle’s medium (DMEM, Sigma Aldrich, Steinheim, Germany) containing 10% fetal bovine serum (FBS; Sigma Aldrich, Steinheim, Germany) and 2 mM l-glutamine (l-Gln, Sigma Aldrich). Cell cultures were incubated at 37 °C in a humidified atmosphere containing 5% CO_2_ up to 30 passages after thawing.

### Viruses

SARS-CoV-2 (isolate MUC-IMB1)^52^ was a kind gift of G. Dobler (Bundeswehr Institute for Microbiology, Germany) and was used in passage 3 after isolation from a patient for infection and passage 4 for titration of nAb titers^23^. SARS-CoV-2 variants B1.617.2 (Delta lineage) or B.1.1.529 (Omicron lineage) were used in passage 5 or passage 3 after isolation from a patient, respectively^53^.

### Syrian golden hamster animal model

All animal experiments were carried out in compliance with the regulations of German animal protection laws and as authorized by the RP Darmstadt and reported according to the ARRIVE guidelines. Six to 12-week-old Syrian golden hamsters (Envigo RMS, Venray, Netherlands) were randomized for sex-matched groups of four animals. Animals were vaccinated intramuscularly (i.m.) in a prime-boost schedule (Day 0 and 21) with 20 μg Comirnaty (BioNTech, Mainz)^27^, 4 μg Spikevax (Moderna)^16^, 1 × 10^10^ vp Jcovden (Johnson & Johnson)^21^ or 0.5 × 10^8^ IU Vaxzevria (AstraZeneca)^33^. These doses were used previously in the respective animal models, exceeding the equivalent human dose based on body weight. Additionally, four animals were vaccinated subcutaneously (s.c.) with 10 µg recombinant SARS-CoV-2 S protein of the original Wuhan lineage (Cat.-No. 40589-V08B1; Sino Biological Europe, Eschborn, Germany) adjuvanted with 500 µg aluminum hydroxide (Allhydrogel adjuvant 2%, vac-alu-250, InvivoGen, San Diego, CA, USA). For approved human vaccines, clinical lots of Comirnaty, Spikevax, and Jcovden were used before the end of shelf-life. For Vaxzevria, a clinical lot was used that had reached the end of shelf-life 20 d or 41 d before the first or the second immunization, respectively. Hamsters inoculated with 100 µl PBS i.m. served as control animals. All vaccinations were performed under transient narcosis with isoflurane (4%) and a final volume of 100 μL vaccine per animal and application. Blood was drawn on days 0, 21, and 35 under transient narcosis with isoflurane (4%). Vaccinated hamsters were challenged on day 35 by applying 4 × 10^3^ TCID_50_ SARS-CoV-2 (isolate MUC-IMB1) of passage 3 in 100 µl volume intranasally under narcosis with ketamin-medetomidine (i.p.: 100 mg/kg body weight ketamine; 0.25 mg/kg body weight medetomidine), antagonized after the manipulations by Atipamezol (s.c.: 0.25 mg/kg body weight). Hamsters were monitored and sacrificed 4 days after infection by overdosing with ketamine-medetomidine (i.p.: 300 mg/kg body weight Ketamin; 0.75 mg/kg body weight medetomidine), and lung tissue was prepared for histology (left lobe), analysis of total RNA (right middle lobe) and titration of live virus (right apical lobe). Table 1

**Table. 1:** Primer and Probe sets used for quantitative qRT-PCR

Primer/Probe	Reference	Sequence
E_Sarbeco_F	Corman et al.^54^	5’-ACAggTACgTTAATAgTTAATAgCgT-3'
E_Sarbeco_R	Corman et al.^54^	5’-ATATTgCAgCAgTACgCACACA-3'
E_Sarbeco probe	Corman et al.^54^	5’-(6FAM)-ACACTAgCCATCCTTACTgCgCTTCg(BBQ)- 3'
RPL18 F	Zivec et al.^56^	5’-gTTTATgAgTCgCACTAACCg-3'
RPL18 R	Zivec et al.^56^	5’-TgTTCTCTCggCCAggAA-3'
RPL18 probe	Zivec et al.^56^	5’-(Cy5)-TCTgTCCCTgTCCCggATgATC(BBQ)- 3'
IL-4-F	Espitia et al.^55^	5’-ACAgAAAAAgggACACCATgCA-3'
IL-4-R	Espitia et al.^55^	5’-gAAgCCCTgCAgATgAggTCT-3'
IL-4 probe	Espitia et al.^55^	5’-(6FAM)-AgACgCCCTTTCAgCAAggAAgAACTCC-(BBQ)-3'
IL-13-F	Espitia et al.^55^	5’-AAATggCgggTTCTgTgC-3'
IL-13-R	Espitia et al.^55^	5’-AATATCCTCTgggTCTTgTAgATgg-3'
IL-13 probe	Espitia et al.^55^	5’-(Cy5)-TggATTCCCTgACCAACATCTCTAgTTgC (BBQ)-3'
IFN-γ-F	Espitia et al.^55^	5’-TgT TgC TCT gCC TCA CTC Agg-3'
IFN-γ-R	Espitia et al.^55^	5’-AAg ACg Agg TCC CCT CCA TTC-3'
IFN-γ probe	Espitia et al.^55^	5’-(Cy5) Tgg CTg CTA CTg CCA ggg CAC ACT C-(BBQ)-3'

*6FAM6* carboxyfluorescein, *BBQ* BlackBerry quencher (BBQ), *Cy5* cyanine 5 fluorophores are indicated.

### Bronchoalveolar lavage (BAL)

Bronchoalveolar lavage (BAL) was performed as previously described^15^ using 2.5 mL PBS applied and excised via the trachea. Cells were harvested by centrifugation (1200 rpm, 4 °C, 5 min) and resuspended in 350 μL TRIzol Reagent (Ambion, Thermo Fisher Scientific).

### Total IgG quantification

Quantification of binding antibodies via ELISA was performed as previously described^15^ using 0.25 μg recombinant unmodified SARS-CoV-2 S protein of the original Wuhan lineage (Sino biologicals) in carbonate buffer (Na_2_CO_3_ 30 mM; NaHCO_3_ 70 mM; pH 9.6) to coat Nunc Maxisorp® 96-well ELISA plates (eBioscience) and HRP-conjugated goat anti-hamster IgG (1:1000 in PBS containing 1% BSA and 0.1% Tween20; Sera care KPL, Cat. 5220-0371) for detection of antigen binding antibodies in the analyzed hamster sera using TMB substrate (eBioscience). After stopping the reaction with 1 N H_2_SO_4_, the absorbance at 450 nm (specific signal) and 630 nm (reference wavelength) was measured.

### Virus neutralization test (VNT)

A virus neutralization test (VNT) was performed as described previously^15,23^. Shortly, 100 TCID_50_ SARS-CoV-2 (MUC-IMB1, B1.617.2 or B.1.1.529) were mixed with 2-fold serially diluted serum samples and incubated at 37 °C for 1 h. Subsequently, the virus-serum mixture was added to 1 × 10^4^ Vero E6 cells seeded in 96-well plates 3 h before. Cells were incubated for 4 days at 37 °C in a humidified atmosphere containing 5% CO_2_. The virus-neutralizing titer was determined by the reciprocal of the highest serum dilution that prevent CPE formation (MUC-IMB1, B1.617.2) or infection determined by immunofluorescence staining (B.1.1.529).

### Immunofluorescence staining assay (IFA)

For the determination of cell cultures infected by SARS-CoV-2 Omicron variant B.1.1.529, the medium of the cells was removed, and the cells were washed with PBS. The cells were then fixed in 4% PFA overnight prior to being permeabilized with 0.5% Triton X-100 for 5 min at room temperature (RT). After washing with PBS, the cells were blocked with 10% goat serum (Abcam, Cat.-No. ab7481) in PBS for 30 min at RT. The cells were then washed and incubated with an anti-SARS-CoV-2 nucleocapsid protein antibody (1:2000; antibodies-online, Cat-No. ABIN6952544) in 2% goat serum in PBS for 1.5 h at RT. After washing with PBS, the cells were incubated with a Cy5-anti-rabbit IgG antibody (1:1000; Invitrogen, Cat-No. A10523) in PBS containing 2% goat serum for 1 h at RT. After a final wash with PBS, fluorescence was assessed under a microscope (ECHO Revolve, ECHO, San Diego, CA, USA).

### Determination of lung virus titers in infected animals

The right apical lobe of the lung was snap-frozen in liquid nitrogen and homogenized in 1 mL ice-cold DMEM containing 2 mM l-Gln and 1% Penicillin/Streptomycin as previously described^15^ using Precellys24 tissue homogenizer (bertin TECHNOLOGIES, Montigny-le-Bretonneux, France) and Lysing Matrix M tubes (MP Bioscience, Hilton, UK). The tenfold serially diluted supernatant was used to inoculate Vero E6 cells for 7 d in a humidified atmosphere at 37 °C. SARS-CoV-2 organ titer was calculated by the TCID_50_ method of Kaerber and Spearman according to virus-induced CPE and normalized to 1 g of homogenized tissue.

### RNA preparation

The right middle lobe of the lung was homogenized in 1 mL TRIzol Reagent (Ambion, Thermo Fisher Scientific) as previously described^15^ using Precellys24 tissue homogenizer (bertin TECHNOLOGIES) and in Lysing Matrix M tubes (MP Bioscience). Cell debris was removed by subsequent centrifugation (6800 rpm; 4 °C; 13 min), and the clear supernatant was used for RNA purification with a Direct-zol RNA MiniPrep kit (Zymo Research, Freiburg (Breisgau), Germany) according to the manufacturer’s instructions.

### Determination of virus genome copy numbers SARS-CoV-2 Egene and hamster RPL18 by qPCR

Quantification of viral RNA in the samples was performed by quantitative reverse transcription-PCR (qRT-PCR) using a Superscript III one-step RT-PCR system with Platinum Tag Polymerase (Invitrogen, Darmstadt, Germany). Primer and probe sequences for SARS-CoV-2 Egene^54^, IL-4^55^, IL-13^55^, IFN-γ^55^, and the RPL18 housekeeping gene^56^ were used as described previously (Table 1).

Each sample was analyzed in triplicates in a 96-well plate format using the CFX96 qPCR cycler (Bio-Rad Laboratories, Hercules, CA) and 5 µl RNA in a total reaction volume of 25 µl. Quantification of the copy numbers of the SARS-CoV-2 E gene was carried out using an internal hamster reference (linear range, 4.5 × 10^6^ to 4.5 × 10^2^ copies^23^). This reference was validated for copy numbers of RPL18 by using a PCR product DNA reference prepared as described previously^57^, and was used for quantification in subsequent runs (linear range, 1.8 × 10^5^ to 1.82 × 10^2^). The following cycling conditions were used: reverse transcription for 600 s at 55 °C, denaturation for 180 s at 94 °C, followed by 45 cycles of 15 sec at 94 °C and 30 s at 58 °C. Quantified E gene copy numbers were normalized to copy numbers of the RPL18 housekeeping gene.

### Histopathological examination

The left lung lobe was fixed in 4% formalin for 7 days. The tissue was subsequently paraffin-embedded, and sections of 4 μm were prepared.

### Hematoxylin-eosin staining

Hematoxylin-eosin staining was carried out in accordance with standard protocols^58^. H&E stained slices were subjected to histopathologic analyses on blinded samples.

### Sirius Red staining

For Sirius Red staining of lung tissue sections we used a modified protocol published by Llewellyn that was used previously^15,59^. First, sections were soaked in Papanicolaous solution 1b Hematoxylin S (Sigma Aldrich) for 2 min and then rinsed with water, ethanol, 3% HCl in ethanol, and 70% ethanol. Sections were then stained in alkaline Sirius red (0.5 g Direktrot 80, Sigma in 50% ethanol containing 0.1‰ NaOH) for 90 min before being rinsed with water. Subsequently, sections were dehydrated with increasing concentrations of ethanol and xylene. Finally, the sections were covered with Entellan (Merck KGaA, Darmstadt, Germany).

### In situ hybridization

To detect viral RNA in the lung tissue sections, fixed paraffin-embedded tissue sections were mounted on glass slides and analyzed by in situ hybridization as described previously^60,61^. The RNAscope® 2.5 HD Assay—RED Kit (Bio-Techne, cat. no. 322360) was used according to the manufacturer’s instructions. To gain access to the target RNA, slides were incubated at 60 °C, deparaffinized with xylene and 100% ethanol, and pretreated with RNAscope® Pretreatment Reagents (cat. no. 322330 and 322000). Subsequently, the RNA-specific probe, targeting the S protein of the SARS-CoV-2 virus (cat. no. 848561), was hybridized to the RNA. After the amplification steps, Fast Red substrate was added to the samples for signal detection. Slides were counterstained with Gill’s Hematoxylin I and 0.02% ammonia water. An RNAscope® Negative Control Probe (cat. no. 310043) was used in parallel to control background staining.

## Supplementary information

Supplementary Material

## Data Availability

All data generated or analyzed during this study are included in this published article (and its supplementary information files).

## Materials availability

Unique biological materials used in this study are available via commercial sources indicated or via the corresponding author of this study upon reasonable request and may require the filing of materials transfer agreements (MTAs) and compensation for shipment.
