# Supplementary material for: Differential efficacy of first licensed western vaccines protecting without immunopathogenesis Wuhan-1-challenged hamsters from severe COVID-19
Source: NPJ Vaccines. 2025 Mar 17;10:51. doi: 10.1038/s41541-025-01100-5 (PMC11914482; doi:10.1038/s41541-025-01100-5)
Supplement: Supplementary file 1 — Supplementary Material [file 41541_2025_1100_MOESM1_ESM.pdf]

SI-Table S1: Histopathological analysis of lung tissue of vaccinated Syrian hamsters upon challenge with SARS-CoV-2. Hamster were vaccinated with medium (MOCK), Alum-adjuvanted Spike protein (Alum+S), Comirnaty, Spikevax, Jcovden or Vaxzevria. The left lobes of vaccinated hamster lungs were dissected 4 dpi with low passage SARS-CoV-2.

Animal No.	Vaccine Group	% Dense Area	Bronchia	Vessels	Dense Area
**124**	**MOCK**	about 20% dense area, bronchial associated	Bronchial epithelium overall only slightly affected, proliferation; little inflammatory infiltration; scattered desquamated cells in the lumen; isolated karyorrhexis; NO bronchial epithelium in the surrounding area	Vascular walls with sporadic inflammatory infiltration; proliferation of the endothelium; sporadic karyorrhexis; hemorrhages	few dense areas, with distinct infiltration by macrophages, lymphocytes, and granulocytes
**128**	**MOCK**	about 40% slightly dense areas, no bronchial association	Bronchial epithelium proliferated, rejected cells in the lumen, mild inflammatory infiltration of the epithelium.	Endothelium of the vessels rounded; otherwise without findings	Areas only moderately condensed, structure of lung still recognizable. Infiltration of macrophages and lymphocytes, no granulocytes, no eosinophils
**140**	**MOCK**	about 50% dense area, not bronchial associated	Proliferation of epithelium; epithelium partially destroyed; sporadic granulocytes in lumen	sporadic granulocytes in the vascular wall, hemorrhages	Areas only moderately compacted, structure of lung still recognizable. Infiltration with macrophages and lymphocytess, no granulocytes, no eosinophils
**143**	**MOCK**	max. 1% dense area	Bronchi only slightly/not affected	Endothelium of the vessels rounded; otherwise without special findings.	Structure of lungs clearly preserved, only minimally dense areas, no macrophages, lymphocytes, many granulocytes, no eosinophils.
**121**	**Alum+S**	about 20% dense areas, bronchial or vascularly associated	Bronchial epithelium with mild inflammatory infiltration; eosinophilic granulocytes in the lumen; few bronchial epithelium in the surrounding tissue	Vascular walls sporadically infiltrated with inflammation; (including eosinphils), sporadic bleeding	few dense areas, with distinct infiltration by macrophages, pneumocytes, lymphocytes and massive infiltration by eosinophilic granulocytes
**125**	**Alum+S**	40% dense area, only partially vascularly associated	no clear bronchi recognizable; massive bronchial epithelium in the surrounding tissue.	Vascular walls clearly affected; infiltration with eosinophils; karyorrhexis; sporadic hemorrhages	One large condensed area; massive infiltration of bronchial epithelium, areas with distinct eosinophils, lymphocytes and marcophages (pneumocytes); areas with distinct karyorrhexis

Animal No.	Vaccine Group	% Dense Area	Bronchia	Vessels	Dense Area
**129**	**Alum+S**	50% dense area, no longer bronchial associated	Bronchial epithelium clearly involved, partially destroyed. Inflammatory infiltration.<br>Granulocytes and eosinophils in the lumen.	Vascular walls clearly affected; infiltration with eosinophils; sporadic distinct hemorrhages	massively condensed areas, infiltration with bronchial epithelium, pneumocytes, macrophages, lymphocytes, granulocytess and many eosinophils
**133**	**Alum+S**	about 50% dense areas, not bronchial associated	proliferation of the epithelium, granulocytes in the lumen	Inflammatory cells in the vascular wall	moderate dense areas; infiltration with pneumocytes, macrophages, lymphocytes, granulocytes and some eosinophils
**122**	**Comirnaty**	Less than 10%, bronchial or vascularly associated	Bronchial epithelium not affected	Vascular walls not affected; perivascular low lymphatic infiltration	minimally dense areas with distinct infiltration by macrophages and lymphocytes; no granulocytes. Very low karyorrhexis.
**126**	**Comirnaty**	max. 5% dense area, bronchial associated	Bronchial epithelium inflammatory infiltrated,<br>some inflammatory cells in the lumen, bronchial epithelium in the surrounding area	Vessels not affected	small dense areas with infiltrated bronchial epithelium, macrophages and lymphocytes, few granuloctes
**130**	**Comirnaty**	no dense areas	Bronchi not affected	Vessels not affected	none
**134**	**Comirnaty**	about 25% dense area, not bronchial associated	Slight proliferation of the epithelium	Rounding of endothelium, mild edema around vessels, hemorrhage	Distinct condensed areas, infiltration with pneumocytes, macrophages, lymphocytes, few granulocytes and a few eosinophils.
**123**	**Spikevax**	about 30 % dense area (many small foci), clearly bornchial and vascular associated.	Bronchial epithelium slightly infiltrated with inflammation; lymphocytes and sporadic eosinophilic granulocytes in the lumen,<br>clearly bronchial epithelium in the surrounding area	Vessel walls sporadically infiltrated with inflammation, (including eosinphils), significant bleedings, clear perivascular infiltrates	clearly dense areas, these clearly infiltrated with macrophages, pneumocytes, lymphocytes, but hardly any granulocytes
**127**	**Spikevax**	max. 10% dense area, strictly bronchial associated	Bronchi not affected	Vessels not affected	small dense areas with infiltrated bronchial epithelium, macrophages and lymphocytes, some eosinophils, sporadic karyorrhexis
**137**	**Spikevax**	max. 1% dense area; only one very small condensed area on a vessel	Bronchi not affected	Vessels not affected	Macrophages, lymphocytes and sporadic eosinophils

Animal No.	Vaccine Group	% Dense Area	Bronchia	Vessels	Dense Area
**141**	**Spikevax**	max. 1% dense area	Bronchi only slightly/not affected	Endothelium of the vessels rounded; otherwise without special findings	Structure of lungs clearly preserved, only minimally dense areas, no macrophages, many lymphocytes, single eosinophils
**131**	**Jcovden**	max. 5% dense area, bronchial associated	Only 1 bronchus affected, with few inflammatory cells in epithelium, exfoliated cells in the lumen	Endothelium of the vessels rounded; otherwise without findings	One distinct dense area; massive infiltration of bronchial epithelium, areas with distinct infiltration of lymphocytes and macrophages (pneumocytes), no granulocytes, no eosinophils, no distinct karyorrhexis
**135**	**Jcovden**	about 40% dense area, not bronchial associated	Only slightly affected, slight focal proliferation of the epithelium	some inflammatory cells in the vascular wall, edema around vessels	Distinct condensed areas, infiltration with a lot of pneumocytes, macrophages, fewer lymphocytes, few granulocytes and some eosinophils
**139**	**Jcovden**	no dense areas	Bronchi not affected	Vessels not affected	none
**144**	**Jcovden**	1% dense area, vascular	Bronchial epithelium in the surrounding area partially destroyed (focal)	Vessels not visibly affected	Structure of lung preserved; only minimal dense areas; many macrophages in infiltration of bronchial epithelium, lymphocytes, no granulocytes, some eosinophils
**132**	**Vaxzevria**	about 1% dense area, bronchial associated	only 1 bronchus clearly affected, proliferating epithelium, exfoliated cells in the lumen	rounded endothelium, inflammatory cells (also sporadic eosinophils) in the vicinity of the vessel	3 small dense areas with lymphocytes, macrophages, granulocytes and eosinophils
**136**	**Vaxzevria**	about 20% dense area, bronchial associated	Only slightly affected, slight focal proliferation of the epithelium	Endothelium of the vessels rounded; otherwise without special findings	Distinct dense areas, infiltration with pneumocytes, macrophages, lymphocytes, few granulocytes and und focal distinct eosinophils
**138**	**Vaxzevria**	about 25% dense area, not bronchial associated	Only slightly affected, slight focal proliferation of the epithelium, but infiltration into the dense areas	Vessels slightly affected	Distinct dense areas; infiltration with pneumocytes, macrophages, lymphocytes, few granulocytes and a few eosinophils
**142**	**Vaxzevria**	about 40% dense area, not bronchial associated	in the dense areas no healthy bronchi bronchi visible; instead, massive infiltration of the epithelium into the surrounding area,<br>blood in the bronchi at the edge.	Vessels not visibly affected, but massive bleeding in the tissue	Dense areas of infiltration with many pneumocytes, macrophages; infiltration of bronchial epithelium, fewer lymphocytes, no granulocytes and no eosinophils

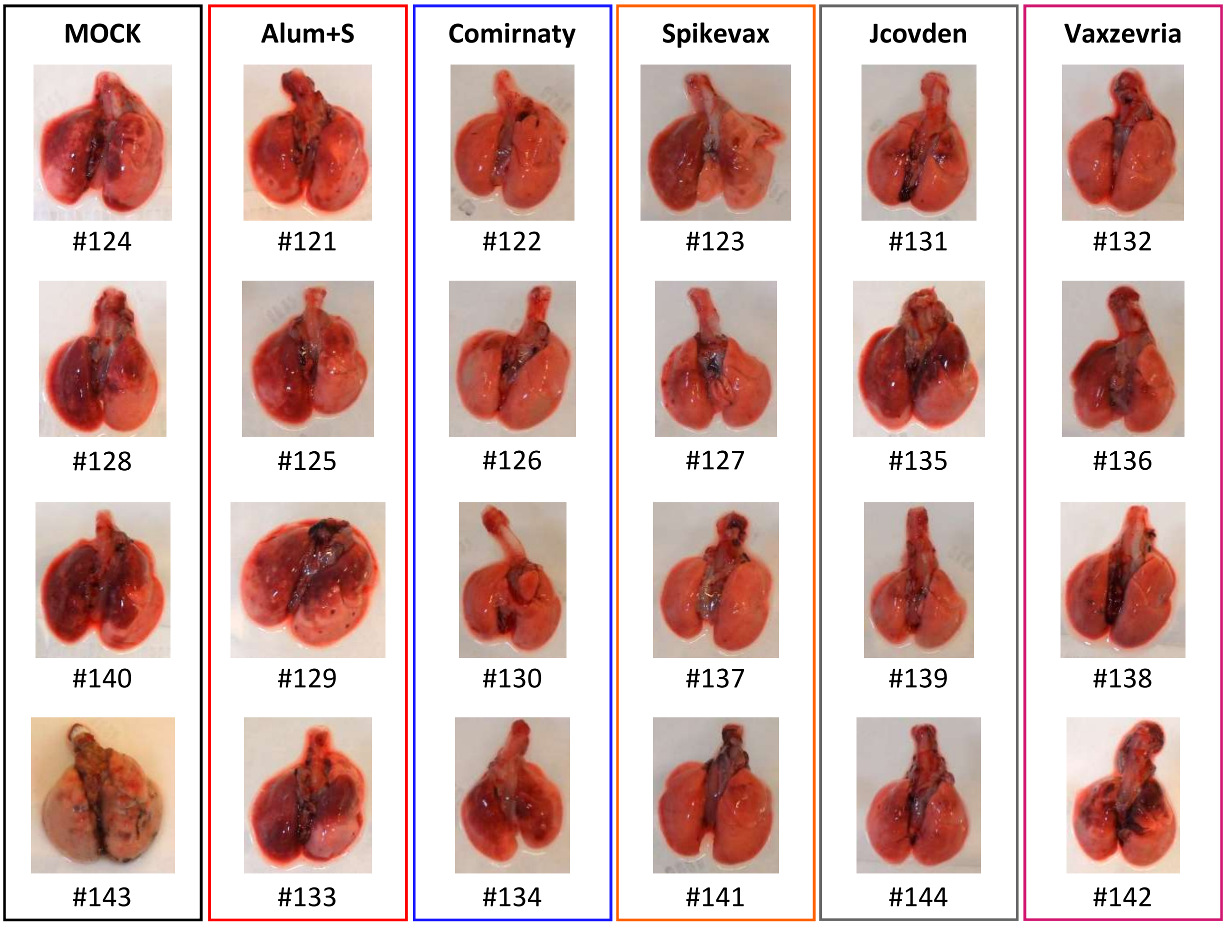

Supplemental Fig. S1. Macroscopic lung pathology. Photographs of hamster lungs prepared 4 dpi, arranged in columns according to the respective vaccine regimen as indicated. Numbers indicate individual animals.

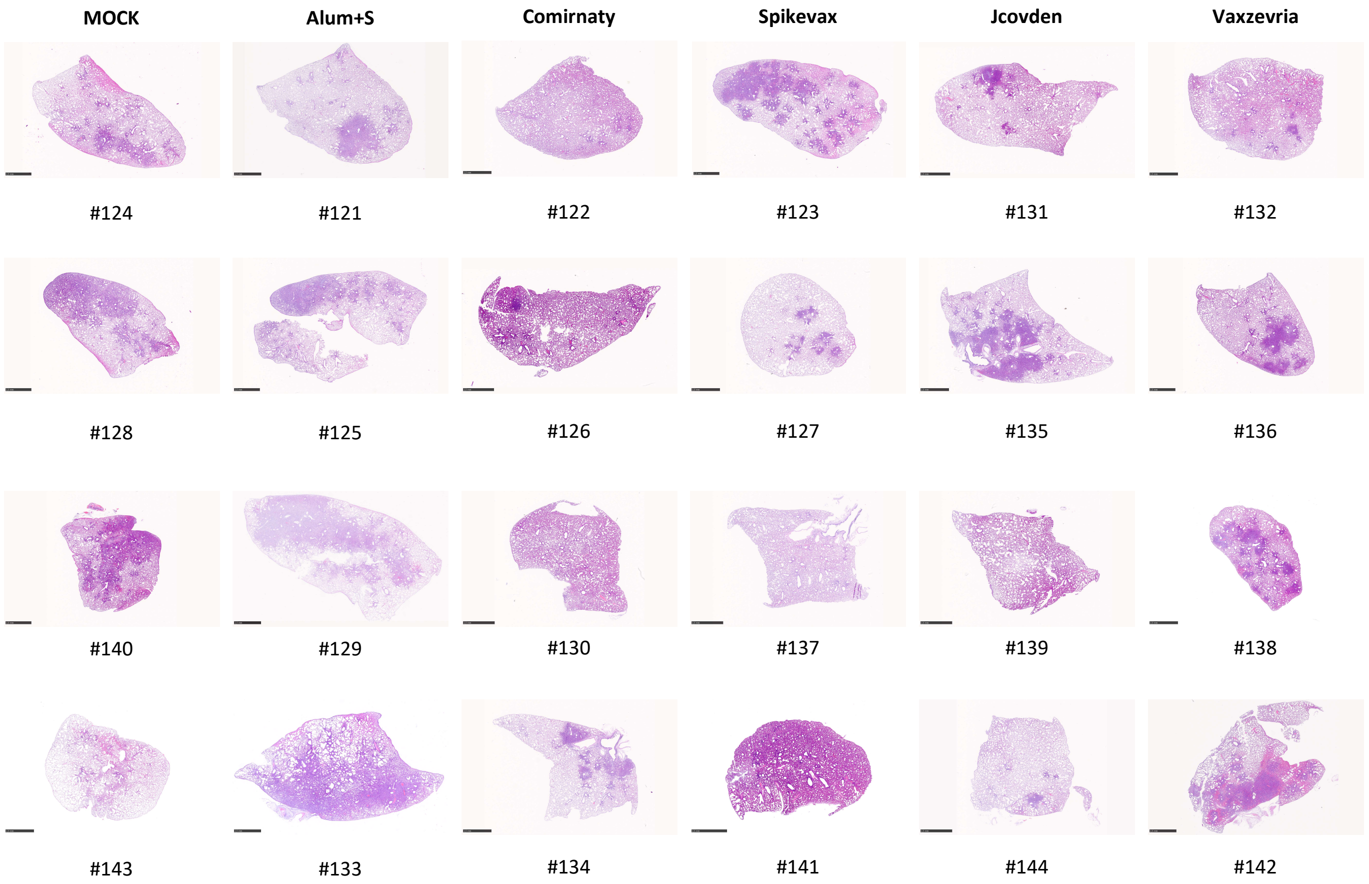

Supplemental Fig. S2. Microscopic lung pathology. Scans of hamster lung slices prepared 4 dpi, arranged in columns according to the respective vaccine regimen as indicated. Lungs were fixed with formalin, embedded in paraffin, and stained with hematoxylin-eosin. Histopathology of lung sections (scale bars: 2,5). Numbers indicate individual animals.

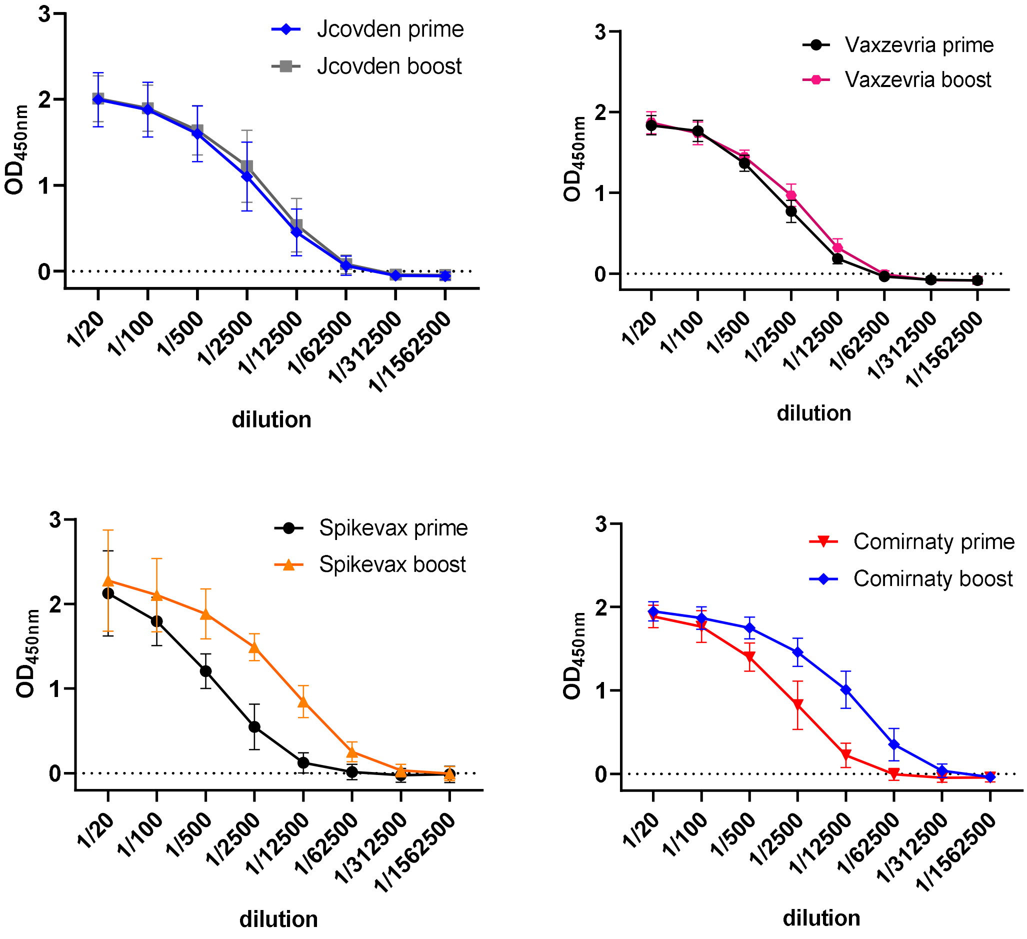

Supplemental Fig. S3. Vaccine-dependent Boosting effect on bAb titers. Sera of immunized hamsters sampled on days 21 (post-prime) and 35 (post-boost) were used for determination of binding antibodies by ELISA. Displayed data have been also used in Fig. 4A, but in re-arranged here for better comparability of immune reactions after one or two immunizations.
